# The Peptide-binding Cavity Is Essential for Als3-mediated Adhesion of *Candida albicans* to Human Cells*

**DOI:** 10.1074/jbc.M114.547877

**Published:** 2014-05-06

**Authors:** Jing Lin, Soon-Hwan Oh, Rhian Jones, James A. Garnett, Paula S. Salgado, Sophia Rusnakova, Steve J. Matthews, Lois L. Hoyer, Ernesto Cota

**Affiliations:** From the ‡ Department of Life Sciences, Imperial College London, Exhibition Road, South Kensington SW7 2AZ, United Kingdom,; the § Department of Pathobiology, University of Illinois Urbana-Champaign, Urbana, Illinois 61802, and; the ¶ Institute for Cell and Molecular Biosciences, Faculty of Medical Sciences, Newcastle University, Newcastle upon Tyne NE2 4HH, United Kingdom

**Keywords:** Adhesion, Aggregation, Candida albicans, Nuclear Magnetic Resonance (NMR), X-ray Crystallography, Als Adhesins, Functional Amyloid, Peptide-binding Cavity, Peptide-binding Protein

## Abstract

The adhesive phenotype of *Candida albicans* contributes to its ability to colonize the host and cause disease. Als proteins are one of the most widely studied *C. albicans* virulence attributes; deletion of *ALS3* produces the greatest reduction in adhesive function. Although adhesive activity is thought to reside within the N-terminal domain of Als proteins (NT-Als), the molecular mechanism of adhesion remains unclear. We designed mutations in NT-Als3 that test the contribution of the peptide-binding cavity (PBC) to *C. albicans* adhesion and assessed the adhesive properties of other NT-Als3 features in the absence of a functional PBC. Structural analysis of purified loss-of-PBC-function mutant proteins showed that the mutations did not alter the overall structure or surface properties of NT-Als3. The mutations were incorporated into full-length *ALS3* and integrated into the *ALS3* locus of a deletion mutant, under control of the native *ALS3* promoter. The PBC mutant phenotype was evaluated in assays using monolayers of human pharyngeal epithelial and umbilical vein endothelial cells, and freshly collected human buccal epithelial cells in suspension. Loss of PBC function resulted in an adhesion phenotype that was indistinguishable from the Δ*als3/*Δ*als3* strain. The adhesive contribution of the Als3 amyloid-forming-region (AFR) was also tested using these methods. *C. albicans* strains producing cell surface Als3 in which the amyloidogenic potential was destroyed showed little contribution of the AFR to adhesion, instead suggesting an aggregative function for the AFR. Collectively, these results demonstrate the essential and principal role of the PBC in Als3 adhesion.

## Teaser

**Background:** Of the eight cell surface glycoproteins in the *C. albicans* Als family, Als3 makes the largest contribution to adhesion to human cells.

**Results:** Mutation of the Als3 peptide-binding cavity (PBC) results in loss of Als3 adhesive function.

**Conclusion:** The PBC is required for Als3 adhesive function.

**Significance:** Interfering with PBC function is a viable strategy for inhibiting *C. albicans* adhesion.

## Introduction

*Candida albicans* is the most common cause of opportunistic mycoses worldwide (1). Among these conditions are oropharyngeal candidiasis that afflicts many HIV-infected patients, vaginal thrush, and denture stomatitis (2–4). *Candida* spp. are among the top four pathogens causing nosocomial bloodstream infections (5), which carry mortality rates ranging between 30 and 40% (1, 6). Cell surface glycoproteins in the *C. albicans* Als family (Als1–Als7 and Als9) are most commonly associated with adhesion of the fungus to host cells, as well as more complex interactions such as invasion and biofilm formation, for which adhesion is a prerequisite (7–9). Deletion of *ALS* genes from *C. albicans* or expression of *ALS* genes in *Saccharomyces cerevisiae* leads to reduction or gain of adhesive function, respectively (10, 11).

The N-terminal domain of Als proteins (NT-Als)^4^ was implicated in adhesive function (12, 13), prompting work to solve its structure (14). X-ray crystallographic analysis of NT-Als9-2 (encoded by an *ALS9* allele) showed that it is characterized by two immunoglobulin-like domains and possesses a peptide-binding cavity (PBC) that can bury up to 6 residues from flexible C termini of polypeptides (14). An invariant Lys (Lys-59) at the end of the PBC recognizes the carboxyl group at the C terminus of a peptide ligand, allowing the remaining peptide backbone to associate in parallel orientation with β-strand G2. In the structure of different NT-Als·ligand complexes, several water molecules anchor polypeptides with different C-terminal sequences in the PBC. These structural observations suggest that the PBC is responsible for Als adhesive interactions and may account for the ability of Als proteins, particularly Als3, to bind to the numerous ligands documented in the literature (*e.g.* 2–4, 12–16).

Here, we describe use of the NT-Als9-2 structure to guide a mutagenesis strategy aimed at disrupting PBC function in Als3. Als3 was selected for this analysis because deleting *ALS3* results in a larger decrease in *C. albicans* adhesion than for any other gene in the family (10). We also targeted the NT-Als C terminus, which contains a conserved amyloid-forming region (AFR) (15) for which adhesive properties have been proposed in the literature (16). We used biophysical techniques to solve the NT-Als3 structure and assessed the structural impact of the site-directed mutations. Finally, we produced the mutant Als3 proteins on the *C. albicans* surface, under control of the native *ALS3* promoter, and tested the strains for their adhesive phenotype in assays using cultured or freshly collected human cells. The complementary information provided from the study of purified proteins using biophysical techniques and phenotypic analysis of mutant proteins displayed in native conditions on the *C. albicans* surface demonstrates the essential contribution of the PBC to Als3 adhesive function.

## EXPERIMENTAL PROCEDURES

### 

#### 

##### DNA Constructs, Protein Expression, and Purification

**FIGURE 1.**
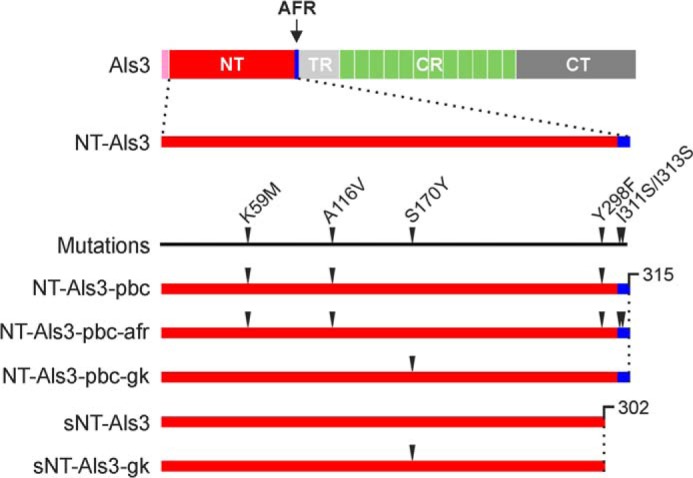
**Als3 constructs and mutants for structural analysis.** *Top*, as a reference, the arrangement of domains in full-length Als3 is shown to scale: the signal sequence (*pink*) is followed by the N-terminal region, containing the amyloid-forming region (*red* and *blue*, respectively), the threonine-rich region (*light gray*), the central repeats (*light green*), and the C-terminal region (*dark gray*). *Bottom*, an expanded N-terminal region is used to show the location of Als3 mutants and the truncation at the C terminus (*CT*) to remove the AFR in sNT-Als3 and sNT-Als3-gk. The numbering of amino acids is consistent with the alignment of mature Als proteins described previously (14).

DNA encoding the N-terminal domain of Als3 (NT-Als3, amino acids 1–315 of the mature protein, KTIT—IVIVA, GenBank Accession No. AY223552), was subcloned in plasmid pET32-Xa-LIC by ligation-independent cloning (Novagen). From this construct, a shorter version of NT-Als3 that excludes the C-terminal AFR (sNT-Als3), was generated by replacing the codon corresponding to Asn-303 with a stop codon. Other mutations that probe the function of the PBC and the AFR in these constructs are summarized in Fig. 1. Mutagenesis was performed with the QuikChange II Kit (Agilent Technologies). Oligonucleotides were designed according to the manufacturer's instructions. Expression and purification of unlabeled and U-^15^N-labeled NT-Als3 constructs/mutants were performed as described for NT-Als1 (17). DNAs were transformed for protein expression in the Origami *Escherichia coli* strain (New England Biolabs) and grown at 37 °C in LB agar plates with 50 μg/ml carbenicillin, 15 μg/ml kanamycin, and 12.5 μg/ml tetracycline. Cultures grown in this medium were clarified by centrifugation at 2000 × *g* and the cell pellets resuspended in 500 ml of LB or M9 minimal medium with 0.07% w/v ^15^N ammonium chloride (Spectra Gases) plus carbenicillin and kanamycin for expression of uniformly labeled ^15^N proteins. Cell cultures at an *A*_600_ 0.5–0.7 were induced with 0.5 mm isopropyl 1-thio-β-d-galactopyranoside and left to grow at 18 °C overnight. Cells were then harvested by centrifugation, resuspended in ice-cold 50 mm Tris, pH 8, 300 mm NaCl plus Complete protease inhibitor (Roche Applied Science), and lysed by French press. The lysate was centrifuged at 30,000 × *g* for 20 min to obtain a cleared supernatant. Soluble thioredoxin-His_6_-Als3 fusions were affinity-purified using nickel-nitrilotriacetic acid resin (Qiagen) in 50 mm Tris, pH 8.0, 300 mm NaCl, 10 mm imidazole, and eluted with 50 mm Tris, pH 8, 300 mm NaCl, 250 mm imidazole. The N-terminal thioredoxin and His_6_ tags were removed from Als3 proteins by Factor Xa (Novagen) and a second nickel affinity purification. Als3 proteins were further purified by size exclusion chromatography on a HiLoad 16/60 Superdex 75 column (GE Healthcare) in 50 mm Tris, pH 8.0, and 300 mm NaCl.

##### NMR Experiments

Samples for NMR experiments were dialyzed extensively against 50 mm sodium phosphate buffer, pH 8.0, 50 mm NaCl and concentrated to 200 μm protein. 10% D_2_O was added to lock the deuterium signal. Proton and ^15^N TROSY-HSQC spectra were recorded at 308 K on a Bruker 800 MHz Avance II spectrometer equipped with a TXI Cryoprobe (Cross Faculty NMR Centre, Imperial College London).

##### Isothermal Titration Calorimetry

ITC experiments were carried out in a VP-ITC MicroCalorimeter (Microcal). Binding isotherms were obtained upon titration of 2.8 mm hepta-Thr (Biomatik) into solutions of 100 μm sNT-Als3 or sNT-Als3-gk. Titrations were conducted in 50 mm sodium phosphate, pH 6.0, and 150 mm NaCl at 303 K. Prior to ITC analysis, protein samples in 3 m urea were dialyzed twice against the buffer above to remove proteins/peptides bound to sNT-Als3 and sNT-Als3-gk during protein purification.

##### Crystallization

sNT-Als3 (10 mg/ml) in 20 mm Tris-HCl, pH 8.0, 100 mm NaCl was incubated with 100-fold molar excess hepta-Thr (sNT-Als3/hepta-Thr) crystallized at 293 K by sitting drop vapor diffusion against 20% v/v PEG 400, 30% w/v PEG 4000, 100 mm sodium citrate, 50 mm ammonium acetate, pH 5.6. NT-Als3-pbc (10 mg/ml) in 20 mm Tris-HCl, pH 8, 100 mm NaCl was crystallized at 293 K by sitting drop vapor diffusion against 25% w/v PEG 4000 and 30% v/v ethylene glycol. sNT-Als3/hepta-Thr crystals were briefly washed in paraffin oil before flash freezing in liquid N_2_. Crystals of NT-Als3-pbc were flash frozen straight in liquid N_2_.

##### X-ray Data Collection, Structure Solution, and Refinement

Data for sNT-Als3/hepta-Thr were collected at 100 K on station I04-1 of the Diamond Light Source, UK. Data were processed using XDS (18) and then scaled with SCALA (19), within XIA2 (20). Initial phases were calculated with molecular replacement with NT-Als9-2 (Protein Data Bank ID code 2Y7N) (14) as the search model using MOLREP (21). Model building was carried out in COOT (22), and refinement with TLS groups (23) was done in REFMAC (24), where 5% of the reflections were omitted for cross-validation. The final model contains 1 molecule of hepta-Thr in the asymmetric unit, and all residues could be built except for the C-terminal Arg-302 (sNT-Als3) and the side chains of Lys-1, Lys-75, Lys-106, Lys-132, Lys-148 (sNT-Als3), and Thr-1 (hepta-Thr).

Data for NT-Als3-pbc were collected at 100 K on station I24 of the Diamond Light Source, UK. Data were processed using XDS (18) and then scaled with SCALA (19), within XIA2 (20). The presence of twinning and significant pseudotranslational symmetry (*u* = 0.01, *v* = 0.23, *w* = −0.5) was detected using XTRIAGE (25). Initial phases were calculated with molecular replacement with NT-Als9-2 as the search model using MOLREP (21). Model building was carried out in COOT (22) and refinement with TLS groups (23), NCS, and twin refinement (*a* = 0.49) was done in REFMAC (24), where 5% of the reflections were omitted for cross-validation. The final model contains 4 molecules of NT-Als3-pbc in the asymmetric unit, and all residues could be built except for the C-terminal Thr-313 (all chains) and the side chains of Lys-1, Asn-53, Lys-75, Lys-106, Glu-129, Lys-132, Lys-148, Lys-149, Tyr-203, Lys-219, Glu-231, Glu-278, Arg-288, Phe-298 (chain A); Lys-1, Lys-106, Glu-129, Lys-132, Asp-145, Lys-148, Lys-149, Lys-164, Lys-178, Gln-207, Lys-219, Glu-231, Tyr-285 (chain B); Lys-1, Lys-75, Lys-106, Lys-132, Lys-148, Lys-164, Lys-178, Gln-187, Met-197, Lys-237, Lys-249, Asn-250, Tyr-285 (chain C); and Lys-1, Lys-75, Lys-106, Lys-132, Lys-148, Arg-158, Lys-164, Arg-294 (chain D). Processing and refinement statistics of the final models are in Table 1.

**TABLE 1:** **Crystallographic data and refinement statistics** Numbers in parentheses refer to the outermost resolution shell.

	sNT-Als3/hepta-Thr	NT-Als3-pbc
**Crystal parameters**		
Space group	*P*2_1_	*P*2_1_
Cell dimensions	*a* = 49.4, *b* = 58.2, *c* = 57.2, β = 114.5	*a* = 112.6, *b* = 67.1, *c* = 112.7, β = 103.5

**Data collection**		
Beamline	DLS I04–1	DLS I24
Wavelength (˚)	0.9173	0.9790
Resolution (˚)	1.40–29.09 (1.40–1.48)	3.00–48.33 (3.00–3.08)
Unique observations	53890 (7562)	32262 (2312)
*R*_sym_*^a^*	0.054 (0.179)	0.097 (0.398)
<*I*>/σ*I*	17.8 (7.7)	9.6 (2.7)
Completeness (%)	93.1 (89.9)	97.5 (95.5)
Redundancy	6.6 (6.8)	2.8 (2.8)

**Refinement**		
*R*_work_/*R*_free_ *^b^* (%)	13.6/17.7	22.9/24.2
Number of protein residues	605	1252
r.m.s.d. stereochemistry*^c^*		
Bond lengths (˚)	0.019	0.009
Bond angles (°)	1.762	1.439
Ramachandran analysis*^d^*		
Residues in outlier regions	0	0
Residues in favored regions	97.0%	93.7%
Residues in allowed regions	100%	100%

*^a^ R*_sym_ = Σ|*I*−<*I*>|/Σ*I* where *I* is the integrated intensity of a given reflection and <*I*> is the mean intensity of multiple corresponding symmetry-related reflections.

*^b^ R*_work_ = Σ‖*F_o_*| − |*F_c_*‖/Σ*F_o_* where *F_o_* and *F_c_* are the observed and calculated structure factors, respectively. *R*_free_ = *R*_work_ calculated using ∼5% random data excluded from the refinement.

*^c^* r.m.s.d. stereochemistry is the deviation from ideal values.

*^d^* Ramachandran analysis was carried out using MOLPROBITY (40).

##### Strain Construction

*C. albicans* strains that produce mutant Als3 proteins were made using constructs in a modified version of the plasmid pUL (11). All PCR amplifications used SC5314 genomic DNA as template and proofreading polymerase. Primers ALS3dnF and ALS3dnR (Table 2) were used to amplify a 358-bp fragment downstream of the *ALS3* coding region. The resulting PCR product was digested with SstII-NgoMIV and cloned into identically digested pUL, resulting in plasmid 1765. Primers were synthesized to add a polylinker to plasmid 1765, expanding its 5′ end multiple cloning site to include HindIII-PstI-BamHI-NcoI-XhoI-SphI. The resulting plasmid was named 2303. Primers 3upPstF and 3upBamR were used to amplify a fragment that includes sequences from upstream of the *ALS3* coding region as well as the first 141 nucleotides of *ALS3*. This fragment was digested with PstI-BamHI and cloned into identically digested plasmid 2303, producing plasmid 2329. Another portion of the *ALS3* 5′ domain was amplified using primers 3cdBamF and 3cdNcoR. The product was digested with BamHI-NcoI and cloned into plasmid 2329, yielding plasmid 2370. The remainder of the *ALS3* coding region was amplified using primers 3cdNcoF and 3dnXhoR, digested with NcoI-XhoI, and cloned into plasmid 2370. The resulting plasmid (2386) included the entire *ALS3* coding region, with flanking sequences to direct integration into the *ALS3* locus, and the *URA3* selectable marker. The *ALS3* coding region included BamHI and NcoI sites within the 5′ domain to provide easy swapping of cassettes encoding mutant *ALS3* sequences. Gene fragments encoding the desired mutations were synthesized by Genewiz Inc., digested with BamHI-NcoI, and cloned into plasmid 2386 to replace the wild-type sequence. The DNA sequence of each resulting construct was verified, and the plasmid was digested with PstI-SstI to produce a fragment for transformation of the *C. albicans* Δ*als3/*Δ*als3 ura3/ura3* strain 2311 (13). Methods for transformation and verification of constructs by Southern blotting and PCR were described previously (11). The resulting *C. albicans* strains were also validated by immunolabeling with anti-Als3 monoclonal antibody 3-A5 using published methods (26).

**TABLE 2:** **Oligonucleotide primers used for *C. albicans* strain construction**

Primer	Sequence (5′–3′)
ALS3dnF	CCC CCG CGG AAG AGC CTG CGA CTA TGA ATT G
ALS3dnR	CCC GCC GGC GTT TGG TAA TTA ACA CAT ATT GC
3upPstF	CCC CTG CAG GTT TTG CTA TGC ACG TTC ATA CTT CC
3upBamR	AAG TTG GGG ATC CTG GTC CCT TAT AAT
3cdBamF	AAT ATA AGG GAC CAG GAT CCC CAA CTT GGA ATG CTG TT
3cdNcoR	GTT TTG TCG CGGTTA GGA TCC CAT GGT AA
3cdNcoF	TTA CCA TGG GAT CCT AAC CGC GAC AAA AC
3dnXhoR	CCC CTC GAG CCT TAA ATA AAC AAG GAT AAT AAT GTG
3upPstF	CCC CTG CAG GTT TTG CTA TGC ACG TTC ATA CTT CC
3upFixBamR	AAC AGC ATT CCA AGT TGG GGT TCC TGG TCC CTT ATA AT
3upFixBamF	ATT ATA AGG GAC CAG GAA CCC CAA CTT GGA ATG CTG TT
3cdFixNcoR	GTT TTG TCG CGG TTA GGA TCG AAT GGT AAG GTG GTC AC
3cdFixNcoF	GTG ACC ACC TTA CCA TTC GAT CCT AAC CGC GAC AAA AC
3dnXhoRPlus	CCC CTC GAG CCT TAA ATA AAT AAG GAT AAT AAT GTG ATC AAA CCA C

Because the BamHI and NcoI restriction sites introduced amino acid changes into the NT-Als3 sequence, the entire strain set was reconstructed following PCR mutagenesis to restore the restriction sites to the wild-type sequence. Q5 DNA polymerase was used in all reactions. PCR products were treated with GeneClean (MP Biomedicals) prior to their use as templates in subsequent PCRs.

Using DNA from construction of the original strain set as the template, three different PCRs were performed for each mutant gene. Primers 3upPstF and 3upFixBamR (Table 2) amplified a 340-bp fragment including the *ALS3* upstream and 5′ sequence (product A). Primers 3upFixBamF and 3cdFixNcoR amplified a 900-bp fragment from the *ALS3* 5′ domain (product B). Primers 3cdFixNcoF and 3dnXhoRPlus amplified the remainder of the *ALS3* coding region as well as some downstream sequence (2.4 kb total; product C). Primers 3upPstF and 3cdFixNcoR were used to amplify a 1.24-kb fragment (product D) from a mixed template of products A and B. A mix of products C and D served as template for amplification with primers 3upPstF and 3dnXhoRPlus to generate a fragment (product E; 3.64 kb) encoding *ALS3* with the correct mutant sequence as well as upstream and downstream regions for integration into the *ALS3* locus of strain 2311. Prior to transformation, product E was cloned into pJET1.2 (CloneJET PCR Cloning Kit; Thermo Scientific), and its DNA sequence was verified. Each plasmid was digested with PstI-XhoI and cloned into similarly digested plasmid 2386 (described above). These constructs were digested with PstI-SacI and the resulting fragment transformed into *C. albicans* 2311. Transformant screening and verification by immunolabeling with anti-Als3 3-A5 were as described above. Primers 3upPstF and 3dnXhoRPlus were used to amplify genomic DNA from each candidate strain, and the DNA sequence of each integrate construct was verified. The resulting strains (Table 3) were used in this study.

**TABLE 3:** ***C. albicans* strains used in this work**

*C. albicans* strains	*ALS3* genotype*^a^*	Reference	Comments
CAI12	*ALS3_LA_/ALS3_SA_*	(31)	Encodes two wild-type *ALS3* alleles.
1893	*ALS3_LA_*	(27)	Encodes only *ALS3_LA_*.
1843	Δ*als3/*Δ*als3*	(11)	Complete deletion of both *ALS3* alleles.
3464	Δ*als3/*Δ*als3*::*ALS3_LA_*	This work	Encodes only *ALS3_LA_*.
3465 (Als3-pbc)	Δ*als3/*Δ*als3*::*ALS3_LA_* (K59M/A116V/Y301F)	This work	Mutations in the PBC create the structure of NT-Als3 bound to a ligand, even in the absence of a peptide. The AFR is not free in solution, but associated with the folded portion of the NT-Als3 domains.
3466 (Als3-gk)	Δ*als3/*Δ*als3*::*ALS3_LA_* (S170Y)	This work	A gatekeeper mutation blocks the entrance of the PBC, eliminating ligand binding. The AFR is not affected and remains free in solution.
3467 (Als3-afr)	Δ*als3/*Δ*als3*::*ALS3_LA_* (I311S/I313S)	This work	Mutations abolish the amyloidogenic potential of the AFR, but do not affect the PBC.
3468 (Als3-pbc-afr)	Δ*als3/*Δ*als3*::*ALS3_LA_* (K59M/A116V/Y301F/I311S/I313S)	This work	Combined mutations as in strains 3465 and 3467. PBC and AFR function are abolished.

*^a^ ALS3* alleles are designated as *ALS3_LA_* or *ALS3_SA_* depending on whether they include 12 or 9 copies of the 36-amino acid tandem repeat sequence in the central domain of the protein (11). *ALS3_LA_* alleles (1138 amino acids) are preferred for these experiments because the increased tandem repeat copy number is believed to form a longer, more extended protein that projects the NT-Als3 domain further away from the cell surface, making it more accessible for functional interactions (11).

##### Adhesion Assays

Methods for evaluating *C. albicans* adhesion to cultured human umbilical vein endothelial cells and freshly collected human buccal epithelial cells were published previously (11). Adhesion to cultured FaDu cells was assayed as described previously (27). Assays were conducted at least in replicate on at least 3 different days.

## RESULTS

### 

#### 

##### Effects of the PBC and AFR on Aggregation of Purified NT-Als3

**FIGURE 2.**
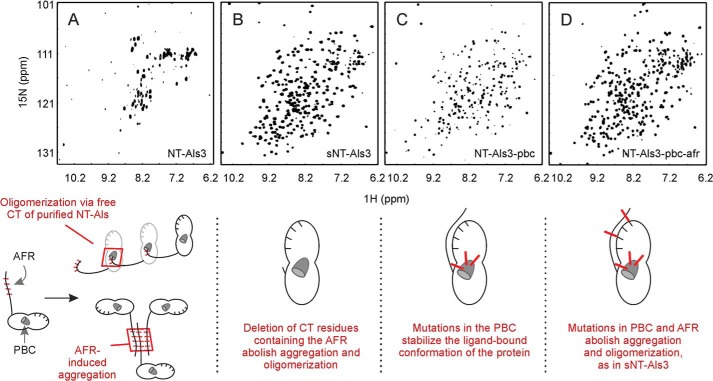
**NMR spectra (^1^H-^15^N TROSY-HSQC) showing the effect of mutations in the PBC and AFR on NT-Als3.** *A*, the NMR spectrum of purified NT-Als3 showed considerably reduced solubility. Two potential mechanisms explain this effect: oligomerization caused by insertion of the free C terminus (*CT*) of the protein as a ligand into the binding pocket of an adjacent NT-Als3 molecule (“self-complementation,” *left*) (14) and aggregation mediated by exposed AFR sequences (30). Note that self-complementation is suppressed in full-length Als proteins on the *C. albicans* surface, where the C terminus is linked to the cell wall via a glycosylphosphatidylinositol anchor remnant (10). *B*, deletion of the AFR resulted in sNT-Als3, a construct free of oligomerization and aggregation effects. *C*, mutations in the PBC (K59M, A116V, and Y301F to generate NT-Als3-pbc) improved protein solubility compared with *A*, presumably due to elimination of self-complementation and decreased aggregation because the AFR was associated with the rest of the NT-Als3 folded structure (Fig. 3). *D*, solubility of the protein in *C* was further improved by removing residual AFR activity via mutations I311S/I313S (NT-Als3-pbc-afr). These results demonstrate at the molecular level the role of the PBC and the AFR in aggregation among purified NT-Als proteins.

Nuclear magnetic resonance (NMR) was used to analyze the structure of NT-Als3 (amino acids 1–315 of the mature protein; amino acids are numbered using a previously described method (14)). A ^1^H-^15^N TROSY-HSQC spectrum of NT-Als3 revealed reduced solubility and broadening of NMR lines beyond signal detection (Fig. 2*A*). Removal of the C-terminal AFR to form sNT-Als3 (amino acids 1–302) resulted in a folded protein that was monomeric in solution, with excellent dispersion of amide resonances (Fig. 2*B*). These observations were potentially attributable to oligomerization caused by NT-Als3 binding of the free C-terminal peptide of adjacent molecules or to aggregation mediated by amyloid-forming regions of the molecules. To distinguish between these possibilities, we designed mutations to interfere with PBC and/or AFR function. Because NT-Als9-2 and NT-Als3 are 68% identical, we used the NT-Als9-2 structure as a reference to design NT-Als3 mutations that create a hydrophobic patch at the end of the PBC, replacing the hydrophilic contacts established with the C-terminal carboxyl group of polypeptides. Specifically, Tyr-301 and Thr-116 side chains that flank Lys-59, positioning its side chain for interaction with ligands (14), were mutated to hydrophobic residues. NMR analysis showed that mutations in NT-Als3-pbc (K59M, A116V, Y301F) shift the conformational equilibrium toward a monomeric, nonaggregated form of the protein that facilitates structural analysis (Fig. 2*C*). Inclusion of two additional mutations (I311S/I313S) was predicted to suppress the amyloidogenic propensity of the AFR, as judged by TANGO analysis (28), and successfully removed the residual NT-Als3 aggregative activity (Fig. 2*D*).

##### A Triple Mutation in the PBC Stabilizes the Ligand-bound Conformation of NT-Als3

**FIGURE 3.**
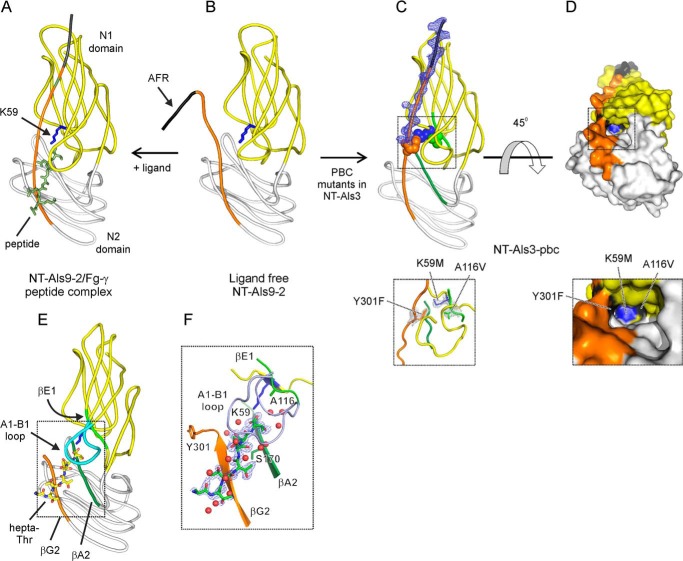
**Crystal structures of NT-Als3-pbc and sNT-Als3.** *A* and *B*, the structures of NT-Als9-2 in complex with Fg-γ peptide and in free form (14) are displayed as a reference to illustrate the effect of mutations in the PBC of NT-Als3. *C*, the crystal structure of NT-Als3-pbc shows a well formed, empty PBC. *Inset below*, the mutated side chains form a hydrophobic patch in the PBC. *D*, surface representation of NT-Als3-pbc is rotated 45° clockwise in the *x* axis relative to *C*. The *inset below* shows the mutated side chains at the end of the PBC. *E*, diagram shows sNT-Als3 in complex with a hepta-Thr ligand. *F*, detail shows interactions between the sNT-Als3 binding site and hepta-Thr. Secondary structure elements that form the binding site are labeled as previously (14) and colored as in *C. Red spheres* represent water molecules. Sigma-A weighted *F_o_* − *F_c_* omit map for these structural models (generated using simulated annealing within PHENIX) were contoured at 3 Å r.m.s.d. electron density around the C terminus and mutated residues in NT-Als3-pbc (*A* and *B*), and at 1.6 Å r.m.s.d. for the hepta-Thr peptide in sNT-Als3. Details of x-ray crystallographic data collection, processing, and structure refinement are in Table 1.

Previous structural analyses showed that the PBC of purified NT-Als adhesins invariably is occupied by a ligand, either exogenously added as in the structure of NT-Als9-2 in complex with a human fibrinogen-γ peptide (NH_3_-GEGQQHHLGGAKQAGDV-CO_2_), or via self-complementation with its own C-terminal strand (Fig. 3, *A* and *B*) (14). The crystallographic structure of NT-Als3-pbc revealed that the mutated residues in the PBC maintained the orientation of the wild-type side chains, but created a hollow, ligand-free cavity (Fig. 3*C*). Unexpectedly, the new interactions in this mutant protein also acted as a “zipper” that propagated the contacts of the flexible C terminus over the surface of the adhesin. Therefore, NT-Als3-pbc recreated the conformation observed in the NT-Als9-2·Fg-γ complex, even in the absence of exogenous ligand. Importantly, the mutated side chains were buried in the PBC, and as a result, NT-Als3-pbc retained surface properties of the wild-type protein (Fig. 3*D*).

##### sNT-Als3, Which Lacks the AFR, Is Competent for Ligand Binding

**FIGURE 4.**
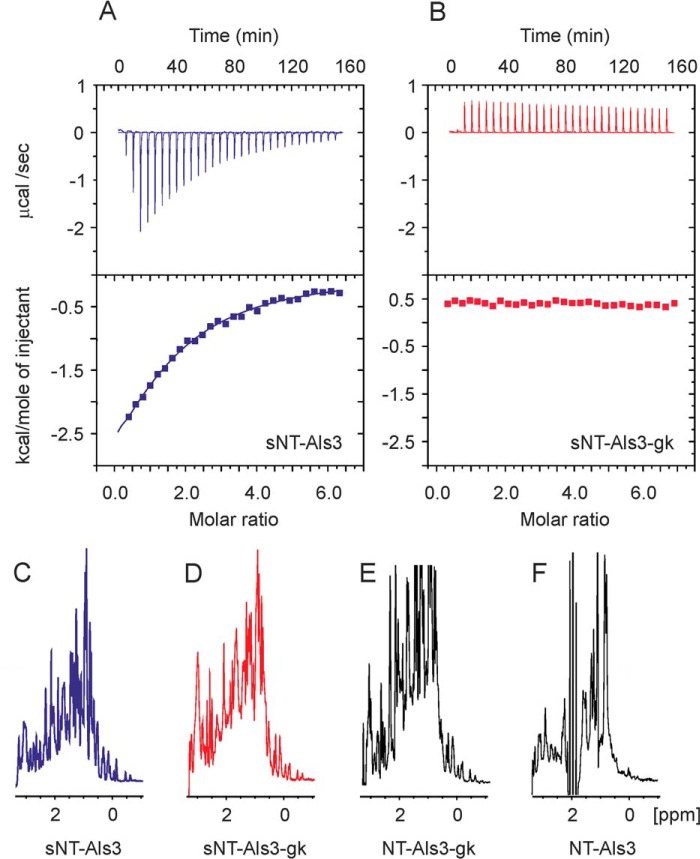
**Biophysical characterization of the S170Y (NT-Als3-gk and sNT-Als3-gk) proteins.** *A*, the interaction of sNT-Als3 and hepta-Thr using ITC is indicated by a reduction of the heat signal as the protein was saturated with ligand. In these conditions, isotherms yielded a low sigmoidicity (or *c* value), precluding the accurate determination of thermodynamic parameters. *B*, sNT-Als3-gk showed no appreciable heats of injection relative to a blank titration, indicating the loss of peptide-binding activity upon mutation. *C* and *D*, the proton spectrum of sNT-Als3-gk (*C*) displays comparable linewidths and dispersion of signals to sNT-Als3 (*D*) in the methyl/methylene region, showing that mutation S170Y does not affect the structure of the protein. *E* and *F*, the NT-Als3-gk fragment, extended with the C-terminal residues containing the AFR (*E*), does not induce the aggregation observed in wild-type adhesin, NT-Als3 (*F*).

The crystal structure of sNT-Als3 (amino acids 1–302) in complex with the model peptide hepta-Thr showed that removal of the C-terminal AFR did not affect the formation of a fully functional PBC (Fig. 3*E*). Notably, the side chain amine of Lys-59 formed a salt bridge with the C-terminal carboxylate of the hepta-Thr ligand, and at least 12 hydrogen bonds between the peptide in extended conformation and this binding cavity were connected via water molecules, resembling the mode of interaction observed in the structure of the NT-Als9-2·Fg-γ complex (Fig. 3*A*). In the residues comprising the immunoglobulin “core” (amino acids 1–299 of the mature protein (14)), this construct had the same overall structure as NT-Als3-pbc, with a r.m.s.d. of 0.9 Å for 296 Cα carbons (DaliLite (29)). When hepta-Thr was bound in the PBC, the Tyr-301 side chain was exposed to the solvent and did not contact Lys-59, possibly from electrostatic repulsion experienced by unpaired charges of the carboxyl group and side chain of Arg-302 at the C terminus. The observation that sNT-Als3 bound hepta-Thr was confirmed in solution by ITC (Fig. 4*A*).

##### A Gatekeeper Mutant (S170Y) Stabilizes the Ligand-free Conformation of NT-Als3

Purified N-terminal fragments of Als5 or small synthetic peptides containing the Als5 AFR induce aggregation of yeast cells that display Als5 on the surface (30). To better define AFR functional contributions, we sought to create NT-Als3 mutant proteins that would separate AFR activity from those of the PBC. At the entrance of the Als3 PBC is Ser-170, a position almost invariably occupied by Ser or Ala among *C. albicans* Als proteins. Mutant S170Y (NT-Als3-gk) was designed as a “gatekeeper” to hinder access of peptides at the entrance of the PBC. Without bound peptide, the C-terminal AFR should be fully flexible and exposed for amyloidogenic interactions. ITC experiments demonstrated that unlike wild-type sNT-Als3, the nonaggregative form of this mutant protein (sNT-Als3-gk, amino acids 1–302) was incapable of binding hepta-Thr (Fig. 4*B*).

Proton NMR spectra showed that the immunoglobulin core of this mutant was folded in solution and comparable with wild-type sNT-Als3 (Fig. 4, *C* and *D*). Unexpectedly, extension of this mutant with the C terminus containing the AFR (NT-Als3-gk, amino acids 1–315) did not lead to aggregation observed in wild-type NT-Als3 (Fig. 4, *E* and *F*, respectively), which indicated that disrupting the function of the PBC greatly decreased *in vitro* self-complementation as a mechanism for aggregation of NT-Als3. Collectively, these results suggested that the S170Y mutant provided a stable, ligand-free form of NT-Als3.

##### The PBC Drives Als3-mediated C. albicans Adhesion

**FIGURE 5.**
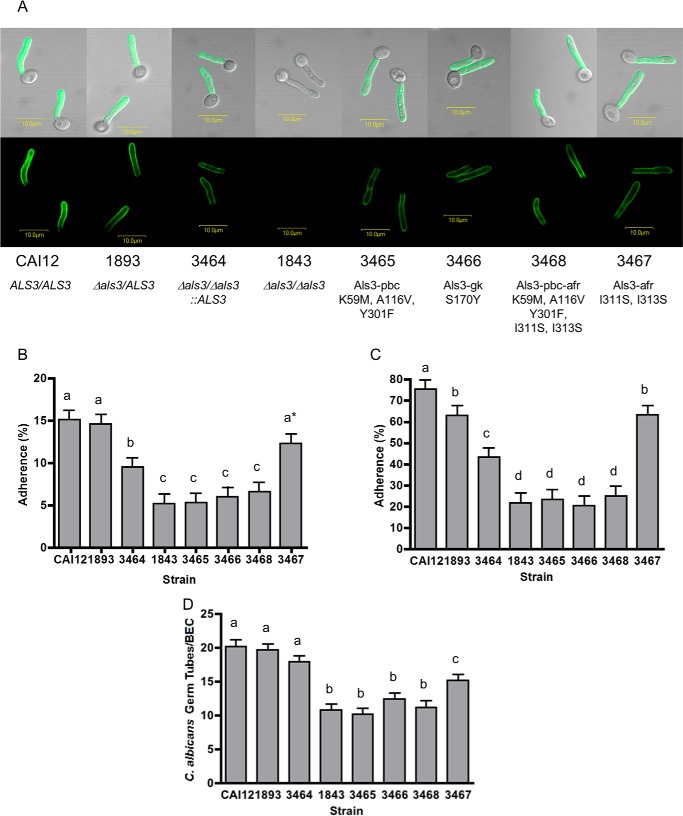
**Adhesion phenotype of *C. albicans* strains displaying mutant Als3 proteins.** *A*, immunofluorescent labeling of *C. albicans* germ tubes with anti-NT-Als3 to visualize display of wild-type or mutant Als3 proteins on the cell surface. Labeling intensity was different between control strains with two *ALS3* alleles (CAI12), one *ALS3* allele (1893), and no *ALS3* alleles (1843). Strains producing mutant proteins Als3-pbc (3465), Als3-gk (3466), Als3-afr (3467), or Als3-pbc-afr (3468) showed labeling intensity similar to their wild-type control strain 3464; however, labeling of each of these strains was less than for strain 1893, which also encoded one *ALS3* allele. *B–D*, incubation of strains lacking PBC function showing significantly decreased adhesion to monolayers of human umbilical vein endothelial cells (*B*), human pharyngeal epithelial cells (FaDu; *C*) or in suspension with freshly collected human buccal epithelial cells (*D*). Results for Als3 lacking only AFR function were more complex, likely reflecting the influence of *C. albicans* cellular aggregation on each adhesion assay (see “Results”). In each histogram, *different lowercase letters* are used to denote strains for which adhesion results are statistically significantly different (*p* < 0.05). In *B*, the *asterisk* indicates that strain 3467 is significantly different from strain CAI12, but not from strain 1893. Results are reported as means ± S.E. (*error bars*).

The site-directed mutations described above were placed into full-length *ALS3* genes and integrated individually into the *ALS3* locus in a Δ*als3/*Δ*als3* background. Visualization of Als3 localization and relative abundance on the *C. albicans* cell surface were accomplished by immunolabeling of the resulting strains with the NT-Als3-specific monoclonal antibody 3-A5 (26). For each strain, distribution of Als3 on the *C. albicans* germ tube surface resembled the control strains (Fig. 5*A*). Relative abundance of Als3 varied depending on whether the strain encoded two *ALS3* alleles (CAI12 (31)), one *ALS3* allele (1893 (27)), or had one allele reintegrated into a null mutant using the cassette for producing the mutant proteins (3464). Strain CAI12 had more Als3 protein than strain 1893; strains 3464 through 3468 exhibited a slight reduction in Als3 protein compared with strain 1893, but showed levels similar to each other (Fig. 5*A*). Each *C. albicans* strain was tested in assays for adhesion to cultured monolayers of human umbilical vein endothelial cells (Fig. 5*B*) and FaDu cells, Fig. 5*C*), as well as freshly collected buccal epithelial cells (BECs; Fig. 5*D*). Because strain 3464 was the most closely related to the strains producing mutant Als3 proteins, it was the best control for interpretation of the adhesion assay data. In all adhesion assays, strains with a mutated PBC were statistically indistinguishable from the null (deletion) mutant 1843. These results indicated that the PBC was essential for Als3-mediated adhesive function, without adhesive contribution from the AFR.

Adhesion assay data suggested an effect of protein abundance on the assay results. Strain CAI12 (with two *ALS3* alleles and the greatest abundance of Als3) consistently showed the greatest amount of adhesion. Loss of an *ALS3* allele led to less Als3 and a reduction in adhesion, with a notable difference occurring between strains 1893 and 3464. Both of these strains encoded the same *ALS3* allele, suggesting that the difference in protein abundance was attributable to integration of the cloning cassette into the *ALS3* locus of strains 3464 through 3468. The effect was assay-dependent with the least effect observed in the BEC assay and a more pronounced change noted in the monolayer assays.

##### The Role of the Als3 AFR Is More Aggregative Than Adhesive

**FIGURE 6.**
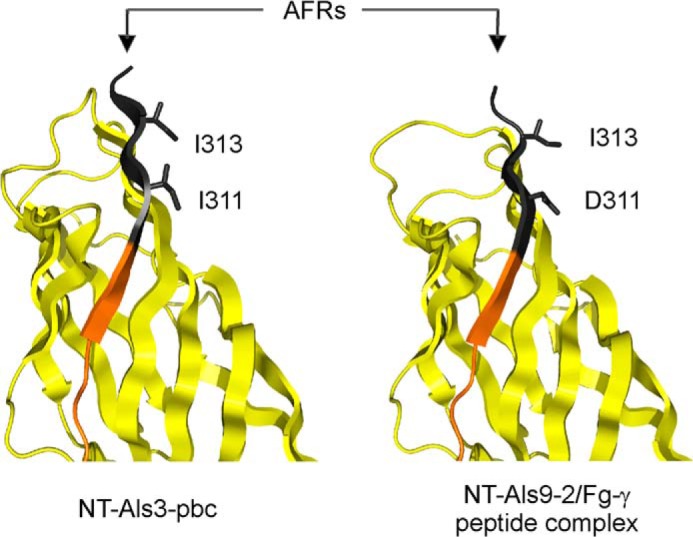
**Position of amino acids substituted to abolish AFR function.** The side chains of residues Ile-311 and Ile-313 were exposed to solvent in the structure of NT-Als3-pbc (*left*), which recreated the conformation of the ligand-bound form of the protein, as shown in the structure of NT-Als9-2 in complex with Fg-γ peptide (14) *right*). For comparison, the equivalent residues in NT-Als9-2 (Asp-311 and Ile-313) are shown. Substitution of Ser for each Ile residue in NT-Als3 eliminated the amyloidogenic propensity of the AFR, as predicted by TANGO (28).

Mutations that abolish the amyloidogenic potential of the AFR were created to test the contribution of the AFR to Als3 function in the adhesion assays. The NT-Als3-pbc structure revealed that the side chains of Ile-311 and Ile-313 in the AFR were exposed to the solvent and did not stabilize the interaction of the C-terminal β-strand with the NT-Als surface (Fig. 6). Double mutant I311S/I313S (NT-Als3-afr) was predicted to suppress the amyloidogenic potential of the AFR, as judged by TANGO analysis (28) and, when incorporated into the NT-Als3-pbc sequence (K59M, A116V, Y301F, I311S, I313S; NT-Als3-pbc-afr), removed the residual aggregative tendency of the protein (Fig. 2, *C* and *D*).

Als3-afr and Als3-pbc-afr were integrated into *C. albicans* to test their adhesion phenotype. Strain 3468 (Als3-pbc-afr) was statistically indistinguishable from strain 3465 (Als3-pbc) in all assays (Fig. 5), suggesting that in the absence of PBC function, AFR activity is not sufficient to affect adhesion assay results. Phenotypic observations for strain 3467 (Als3-afr) were more complex. In the human umbilical vein endothelial cells and FaDu monolayer assays, 3467 showed increased adhesion relative to the control strain (3464), suggesting that the AFR inhibited Als3 adhesion. This effect may occur if the AFR caused aggregation between Als3 molecules on the *C. albicans* surface. As a result, we hypothesize that a decrease in the aggregative potential of the AFR by the I311S/I313S mutant made the PBC more accessible for interactions with host cell ligands. Germ tubes for these assays were grown statically and transferred to cell culture wells containing the host cell monolayer to test the adhesive phenotype. Microscopic inspection of the cell culture wells indicated that germ tubes adhered individually in this static assay. In contrast, results for the BEC assay showed decreased adhesion of strain 3467 (Als3-afr) relative to the control strain (3464), suggesting that the AFR promoted adhesion. Germ tubes for this assay were grown in a shaking flask, to which the BECs subsequently were added and co-incubated. Adhesion was evaluated by microscopy, counting germ tubes in direct contact with a BEC as adherent. In contrast to the monolayer assays (above), germ tubes in the BEC assay formed aggregates that adhered to the human cells. Each aggregate brought many germ tubes into close proximity with the BEC, providing the potential for a higher count of “adherent” cells. Germ tubes of strain 3464 that adhered to BECs were significantly more aggregated than those of strain 3467 (7.7 ± 0.7 cells/aggregate compared with 5.2 ± 0.7, *p* < 0.05). In addition, although not statistically significant, more germ tubes of strain 3467 adhered to BECs individually (mean = 40.3 ± 2.8 of 100 adhesion events) than germ tubes of strain 3464 (29.3 ± 2.8; *p* = 0.1). Collectively, these observations were consistent with the conclusion that the Als3 AFR mediated interactions between Als molecules on the same *C. albicans* cell, as well as between *C. albicans* cells, contributing aggregative, rather than adhesive, effects. These data also illustrated the contribution of aggregative interactions to commonly used assays for evaluating *C. albicans* adhesion.

##### Sequence-based Structural Predictions and Als Ligand-binding Specificity

**FIGURE 7.**
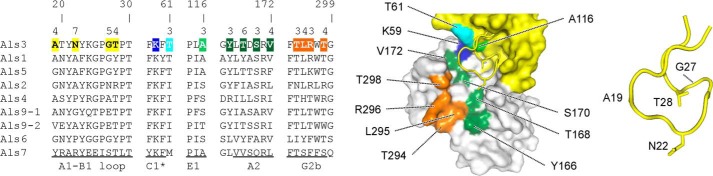
**Alignment of NT-Als PBC residues with side chains that interact with peptide ligands.** The structures of NT-Als9-2 and NT-Als3 allowed prediction across the Als family of amino acids with side chains in close proximity to ligands in the PBC. These residues are *highlighted* in the Als3 sequence (*left*) and include residues 19, 22, 27, and 28 in the A1-B1 loop that act as a “lid” over the binding cavity; residues 61 and 116 (β-strands C1* and E1, respectively) that flank the invariant Lys-59 (*highlighted* in *blue*); residues 166, 168, 170, and 172 in the A2 β-strand; residues 294, 295, 296, and 298 in the G2b β-strand. *Numbers* on *top* of the alignment show the substitutions observed in the family, with positions 22 and 168 containing the greatest sequence diversity. Residues *highlighted* in the alignment are mapped in the sNT-Als3 structure (*center*). For clarity, relevant residues in the A1-B1 loop are shown separately (*right*).

Data presented here demonstrated the principal role of the PBC in Als adhesion, an initial step toward a mechanistic understanding of Als function. The PBC-mediated adhesive mechanism can explain the ability of Als proteins to bind a wide variety of peptide ligands. Work presented here provided a second high resolution NT-Als structure and prompted an examination of amino acids with side chains predicted to interact with ligands in the PBC of the other Als proteins (Fig. 7). Barring significant changes in the NT-Als protein backbone conformation, variable amino acids with side chains in close proximity with ligands should modulate changes in binding specificity across the Als family. Of note, NT-Als1 and NT-Als3 (the most conserved pair of NT-Als proteins with 83% identity in this region) differed in positions 22, 28, and 168, suggesting that their individual ligand-binding specificities can be transferred from one adhesin to another with a minimal number of amino acid substitutions. In contrast, NT-Als7 had 45% amino acid identity with NT-Als3 and 11 changes in this amino acid subset. Future experiments will focus on exploring these relationships, which are expected to further verify the importance of the PBC to Als adhesive function, and define functional interrelationships among the family of Als proteins.

## DISCUSSION

Previous studies led to the solution of the NT-Als9-2 structure that revealed a PBC and proposed a novel mechanism for the ability of Als proteins to bind a broad variety of protein ligands (14). In the current work, we refined our knowledge of NT-Als structure by extending crystallographic analysis to NT-Als3. We used structurally informed mutagenesis to remove essential contacts for ligand binding (NT-Als3-pbc) or to exclude ligand entry into the PBC (NT-Als3-gk), without affecting NT-Als3 surface structure. We also used amino acid substitutions to eliminate the amyloidogenic potential of the AFR region of the protein (NT-Als3-afr). Structural analysis of these mutant proteins revealed previously unrecognized interplay between the PBC and AFR, in which ligand-binding status of the PBC affects AFR placement relative to the NT-Als3 protein domains. These mutations were placed into the context of full-length Als3 and displayed in their native context on the *C. albicans* surface. Resulting strains were assayed in widely used adhesion models and successfully dissected the effects of *C. albicans* attachment to human cells (here defined as “adhesion”), from *C. albicans* intercellular interactions (here, defined as cellular “aggregation”).

Adhesion, as evaluated in these standard models, likely is often considered at face value, without contemplating the many complex interactions that are reflected in the final data. In the *C. albicans* Als literature, the adhesive phenotype is often evaluated using systems that involve protein overproduction in nonnative settings. These approaches allow the study of an individual adhesin away from the naturally sticky context of the *C. albicans* cell. Amid these approaches, then, our results are stunning: substitution of one or three amino acids is sufficient to change *C. albicans* adhesive phenotype from wild-type to that of a Δ*als3/*Δ*als3* deletion mutant, while still presenting the native surface of Als3 on the fungal cell. The ability of Als3 PBC function to emerge so clearly from the complex interactions of the adhesion assay is a testament to the importance of Als3 in *C. albicans* adhesion and the importance of the PBC in the adhesion event.

Initial speculation about the Als ligand-binding mechanism suggested that it occurs by interactions between the NT-Als surface and the surface of the ligand (8, 16, 32). This idea became more unlikely, however, as numerous Als ligands, particularly for Als3, were reported in the literature. Cell biology-based inquiry has provided an extensive list of divergent binding partners for Als3 including human fibronectin, laminin, collagen, gp96, EGFR, HER2, N-cadherin, E-cadherin, fibrinogen, casein, equine ferritin, bovine serum albumin, and *Streptococcus gordonii* SspB (8, 9, 32–37). These reports raise the question of how NT-Als3 can adapt to surfaces of so many structurally unrelated ligands to establish biologically relevant interactions. Solution of the NT-Als molecular structure and discovery of the PBC (12) provided the means to reconcile these data into a model of Als protein structure and function that is supported by experimental techniques able to provide information with atomic resolution. That model was further tested here by site-directed mutagenesis of the PBC and accompanying structural analyses demonstrating that the mutations do not alter surface structure of the protein. Loss of adhesive function for the Als3-pbc protein shows that Als3 binds free C termini of host proteins and excludes the idea that Als3 interacts with ligands using surface-surface interactions.

**FIGURE 8.**
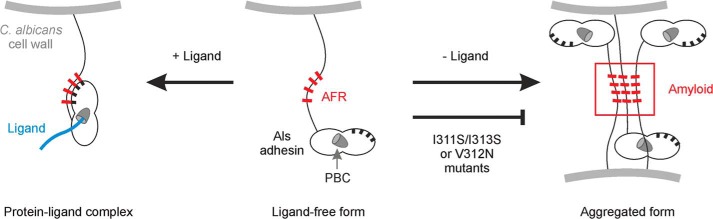
**Proposed conformations of the AFR in Als adhesins.** Newly synthesized Als3 in “free form” (*center*), is competent for ligand binding via the PBC or for aggregation mediated by the AFR. As inferred from Garcia *et al.* (30) and our NMR data (Fig. 2), interaction between the AFR of Als3 proteins on different *C. albicans* cells leads to formation of aggregates (amyloid; *right*). Mutations in this region (*e.g.* V312N (30) or I311S/I313S (Fig. 2)) abolish this phenotype. In the presence of ligands, the AFR attaches to the surface of the adhesin (*left*), as revealed by the crystal structure of the NT-Als9·Fg-γ complex (14). High affinity ligands are predicted to shift the equilibrium toward this nonaggregative protein-ligand complex. If aggregative interactions are disrupted by mutation of the AFR, the PBC could become more available to bind ligands, as evidenced by the increased binding to human cell monolayers of the Als3-afr strain (Fig. 5).

Experiments presented here provide the first data to address function of the Als PBC. In contrast, function of the AFR has been discussed extensively in the *C. albicans* literature and a functional model proposed (16). This model features adhesive contact between the NT domains of mature Als molecules and a mechanical stimulus to stretch each Als molecule, causing exposure of previously obscured amyloid-forming sequences. It is proposed that interaction between these sequences leads to amyloid nanodomain formation that induces strong adhesive interactions. Some of our data are in agreement with this model whereas others suggest the need for modifications. Our results demonstrate the principal role of the PBC in ligand binding and recognition of cell surfaces and do not support a role for the AFR, in a free or clustered state, to perform these functions. Importantly, structural information shows that the AFR is located at the boundary between the NT and the T-rich domains, not in the T-rich domain as suggested previously (16). This region is competent for amyloidogenic interactions in the ligand-free form of the protein, but it is docked to the surface of the adhesin in the ligand-bound form, meaning that ligand binding (*e.g.* adhesion) and AFR-mediated amyloid formation (involved in *C. albicans* aggregation) are mutually exclusive processes (Fig. 8). These data also show that ligand binding by the PBC is passive, *i.e.* does not require activation of Als domains by mechanical/extension forces (16).

Nonetheless, our data demonstrating that germ tubes aggregate when grown with shaking, but not in static culture, support the concept of Als protein force-activated aggregative interactions. Similarly, our phenotypic data for Als3-afr in adhesion assays suggest that the AFR mediates interactions between Als proteins on the same *C. albicans* cell or between Als proteins on different *C. albicans* cells, consistent with the previous model.

Strains 1893 and 3464 encode the same *ALS3* allele, yet can differ significantly in their adhesion phenotype in an assay-specific manner (Fig. 5). The only apparent difference between these strains is the abundance of Als3 cell surface protein, as detected by immunofluorescent labeling with an anti-Als3-specific monoclonal antibody. This demonstration of the effect of protein abundance on adhesion assay conclusions raises new questions regarding the pre-eminence of Als3 contribution to *C. albicans* adhesion: does deletion of *ALS3* cause the largest decrease in *C. albicans* adhesion because Als3 is an adhesin with the greatest affinity to ligands, or because Als3 is produced in abundance across the greatest expanse of the *C. albicans* germ tube? Other Als proteins are known to have a restricted localization on the *C. albicans* germ tube (*e.g.* Als4 (38)) or to be present at such low abundance that they cannot be visualized using standard immunolabeling techniques (*e.g.* Als5, Als6 (26, 39)). Consequently, standard adhesion assays suggest little contribution of these proteins to *C. albicans* phenotype. It is highly likely that misexpression of ALS genes to increase their abundance to native Als3 levels and/or alter the cell surface distribution of the encoded proteins may result in different conclusions regarding adhesive function of the various Als proteins.

The *C. albicans* strains described here provide the means to dissect the effects of the PBC and AFR on more complex phenotypes important to *C. albicans* pathogenesis, such as invasion and biofilm formation. Our suite of tools and approaches are also poised to address questions regarding the relationship between Als protein sequence, localization, and abundance to *C. albicans* phenotype. These data will contribute additional insight into the mechanism of Als protein function.

## Footnotes

4 The abbreviations used are:

NT-Als: N-terminal domain of Als proteins
AFR: amyloid-forming region
BEC: buccal epithelial cell
FaDu: human pharyngeal epithelial cell
Fg-γ: fibrinogen-γ
gk: gatekeeper
ITC: isothermal titration calorimetry
PBC: peptide-binding cavity
r.m.s.d.: root mean square deviation
sNT-Als3: shorter version of NT-Als3 that excludes the C-terminal AFR.
